# Popular Nutrition-Related Mobile Apps: An Agreement Assessment Against a UK Reference Method

**DOI:** 10.2196/mhealth.9838

**Published:** 2019-02-20

**Authors:** Rosalind Fallaize, Rodrigo Zenun Franco, Jennifer Pasang, Faustina Hwang, Julie A Lovegrove

**Affiliations:** 1 Hugh Sinclair Unit of Human Nutrition and Institute for Cardiovascular and Metabolic Research Department of Food and Nutritional Sciences University of Reading Reading United Kingdom; 2 Department of Biomedical Engineering School of Biological Sciences University of Reading Reading United Kingdom

**Keywords:** weighed food records, smartphone application, dietary assessment, diet apps, nutrition apps, diet records, mobile applications

## Abstract

**Background:**

Nutrition-related apps are commonly used to provide information about the user’s dietary intake, but limited research has been performed to assess how well their outputs agree with those from standard methods.

**Objective:**

The objective of our study was to evaluate the level of agreement of popular nutrition-related apps for the assessment of energy and available macronutrients and micronutrients against a UK reference method.

**Methods:**

We compared dietary analysis of 24-hour weighed food records (n=20) between 5 nutrition-related apps (Samsung Health, MyFitnessPal, FatSecret, Noom Coach, and Lose It!) and Dietplan6 (reference method), using app versions available in the United Kingdom. We compared estimates of energy, macronutrients (carbohydrate, protein, fat, saturated fat, and fiber), and micronutrients (sodium, calcium, iron, vitamin A, and vitamin C) using paired *t* tests and Wilcoxon signed-rank tests, correlation coefficients, and Bland-Altman plots. We obtained 24-hour weighed food records from 20 participants (15 female, 5 male participants; mean age 36.3 years; mean body mass index 22.9 kg/m^2^) from previous controlled studies conducted at the Hugh Sinclair Unit of Human Nutrition, University of Reading, Reading, UK. Participants had recorded their food consumption over a 24-hour period using standard protocols.

**Results:**

The difference in estimation of energy and saturated fat intake between Dietplan6 and the diet apps was not significant. Estimates of protein and sodium intake were significantly lower using Lose It! and FatSecret than using Dietplan6. Lose It! also gave significantly lower estimates for other reported outputs (carbohydrate, fat, fiber, and sodium) than did Dietplan6. Samsung Health and MyFitnessPal significantly underestimated calcium, iron, and vitamin C compared with Dietplan6, although there was no significant difference for vitamin A. We observed no other significant differences between Dietplan6 and the apps. Correlation coefficients ranged from *r*=–.12 for iron (Samsung Health vs Dietplan6) to *r*=.91 for protein (FatSecret vs Dietplan6). Noom Coach was limited to energy output, but it had a high correlation with Dietplan6 (*r*=.91). Samsung Health had the greatest variation of correlation, with energy at *r*=.79. Bland-Altman analysis revealed potential proportional bias for vitamin A.

**Conclusions:**

The findings suggest that the apps provide estimates of energy and saturated fat intake comparable with estimates by Dietplan6. With the exception of Lose It!, the apps also provided comparable estimates of carbohydrate, total fat, and fiber. FatSecret and Lose It! tended to underestimate protein and sodium. Estimates of micronutrient intake (calcium, iron, vitamin A, and vitamin C) by 2 apps (Samsung Health and MyFitnessPal) were inconsistent and less reliable. Lose It! was the app least comparable with Dietplan6. As the use and availability of apps grows, this study helps clinicians and researchers to make better-informed decisions about using these apps in research and practice.

## Introduction

### Background

The advancement of technology has led to the development of novel electronic dietary assessment methods, for example, in the form of nutrition-related apps, which are commonly used on mobile phones. These apps provide information about the user’s overall energy intake and expenditure. Benefits that arise from this method are the ease of use, convenience, and logging of food in real time [1,2], and the wide availability of these apps via downloading from app stores. The apps may also enable greater self-monitoring by individuals with chronic diseases such as obesity, cardiovascular disease, and type 2 diabetes [3-5], contributing to nutrition care. However, this form of dietary assessment has yet to be validated and wholly accepted by health care providers to confidently recommend its use. Known limitations of these apps include limited nutrient data, particularly for micronutrients, and inaccurate nutrient compositions [1,6]. Despite this, reported nutrition app use in dietetic practice in the United Kingdom, New Zealand, and Australia (5% response rate from the practitioners contacted) is high (62%) [7].

To date, limited research has been performed on nutrition-related apps, whether it be commercial or researcher developed, and hence the lack of standard methods for assessing validity and accuracy [8]. Furthermore, with the growing number of commercial nutrition-related apps, the difficulty of creating criteria to assess them increases with varying features and databases. This is often exacerbated by a lack of information on the source of nutritional data used by apps. Previous studies by Carter et al [6] and Raatz et al [9] comparing commercial nutrition-related apps with reference methods have used 24-hour recalls and weighed food records (WFRs), respectively. Both studies found no significant differences in mean energy and macronutrients between the nutrition-related app and the reference method. Carter et al used an app for mobile phones called My Meal Mate [6], whereas Raatz, et al used Tap & Track [9]. However, My Meal Mate is not considered a “popular” mobile phone app (with fewer than 500,000 installs), as identified by Franco et al [10], and Tap & Track is available only in Apple Inc’s App Store, limiting its use.

### Objective

Exploring the accuracy of popular apps would ensure that the findings from studies that used these apps are relevant for the commercial market. Thus, the aim of this study was to evaluate the extent to which popular nutrition-related apps give energy, macronutrient, and micronutrient data comparable with the research standard method using Dietplan6 version 6.0 (Forestfield Software Ltd) analysis program, using 24-hour WFR as an input.

## Methods

### Dietary Assessment

We obtained 20 handwritten 24-hour WFRs from a previous controlled diet study conducted at the Hugh Sinclair Unit of Human Nutrition, University of Reading, Reading, UK. Participants (female, n=15; male, n=5; mean age 36.3 years; mean body mass index 22.9 kg/m^2^) had recorded their food consumption over a 24-hour period using standard protocols [11]. Briefly, the WFR consisted of recording the time, brand name, description of food or drink, cooking method, weight (grams), and leftovers (grams) prospectively. We entered the preexisting 24-hour WFRs into 5 nutrition-related mobile apps using a Samsung Galaxy Tab 4 tablet (Samsung Electronics Co, Ltd, Suwon, South Korea) or a Moto G smartphone (Motorola Mobility LLC, Chicago, IL, USA), using the app versions available in the UK Google Play Store (Google LLC, Mountain View, CA, USA) in January 2016. We compared these apps against a reference research method, Dietplan6. We selected the 5 apps based on popularity (a minimum of 500,000 installs from the Google Play Store), availability as a free download, and having a feature to provide energy (kcal) calculations. As defined and reported by Franco et al, the most popular apps meeting these criteria were Samsung Health (S Health; Samsung), MyFitnessPal (MyFitnessPal, Inc), FatSecret (Secret Industries Pty Ltd), Noom Coach (Noom Inc), and Lose It! (FitNow, Inc), which we therefore used in this study. Further details of the features available in these apps have been previously published [10].

### Samsung Health

S Health features tracking of energy, macronutrients, micronutrients (including sodium, calcium, iron, vitamin A, and vitamin C), water (cups per day), and exercise. S Health compares intake data with the recommended intake and color codes the intake as low, average, and high.

### MyFitnessPal

MyFitnessPal features tracking of energy, macronutrients, and micronutrients (including sodium, calcium, iron, vitamin A, and vitamin C). Together with S Health, it has the most extensive nutrient output of all 5 apps. MyFitnessPal compares intake data with the US recommended daily allowance. Tracking for water (cups per day) and exercise is also available. The user can create foods or recipes if they are not available in MyFitnessPal’s database. Foods added by other users can also be selected for data entry [12].

### FatSecret

FatSecret reports energy, macronutrients, and micronutrients (only sodium). It allows the user to add exercise and to save their own meals and add foods not available in the FatSecret database. Recipes added by other users can also be selected. The app compares intake with the recommended intake for macronutrients only.

### Noom Coach

Noom Coach reports only energy intake and compares it with the daily energy recommendation. In addition, exercise can be logged. Recipes can be saved onto the app for future use.

### Lose It!

Lose It! reports energy, macronutrients, and sodium. Similar to the other 4 apps, this app allows exercise to be logged. The app compares intake with the recommended intake. Foods or recipes can be created and shared if they are not available in Lose It!’s own database.

### Dietplan6

Dietplan6 is a nutrition analysis software package for professional dietitians and nutritionists that we used as the reference method in this study. Dietplan6 reports energy, and all macronutrients and micronutrients. The nutrient composition of foods selected was the *McCance and Widdowson’s The Composition of Foods, 6th edition* [13].

### Data Input

For each food in the handwritten WFRs, we selected the same food or a suitable (ie, similar) substitute from the databases of all the apps to ensure consistency in diet entry. Supplements were not included in the WFRs. All data input was completed by a single trained researcher (JP) and verified by a second researcher (RZF).

### Statistical Analysis

We checked the energy and available macronutrient (carbohydrate, protein, total fat, saturated fat, and fiber) and micronutrient (sodium, calcium, iron, vitamin A, and vitamin C) data for normality using the D’Agostino-Pearson test. To explore differences in outputs between the diet apps and the reference method, Dietplan6, we used paired 2-sample *t* tests (2-tailed). We analyzed nonnormally distributed data using the Wilcoxon signed-rank test. We used Pearson correlations for normal distributions and Spearman correlation for nonnormally distributed data. We also analyzed data using Bland-Altman plots and correlation coefficients (*r*). Bland-Altman analysis examined the agreement between 2 samples using the standard deviation and mean to assess the linear relationship of the variables. We analyzed data using the SciPy 1.1.0 Stats package for Python [14], and we considered a *P* value smaller than .01 (Bonferroni correction applied based on the initial *P* value of .05) to be significant for hypothesis tests and correlation significances.

## Results

### App Outputs

All of the popular diet apps provided outputs for energy (kcal). With the exception of Noom Coach, outputs were also provided for carbohydrate (g), protein (g), fat (g), fiber (g), and sodium (mg). Additionally, S Health, MyFitnessPal, and Lose It! gave outputs for saturated fat (g). Of the 5 apps tested, only 2 apps had micronutrient outputs other than sodium (calcium, iron, vitamin A, and vitamin C): S Health and MyFitnessPal.

### Comparison of Energy and Nutrient Intake Between Dietplan6 and the Apps

Multimedia Appendix 1 shows individual comparisons of energy and nutrient analysis between Dietplan6 and popular diet apps. We observed no significant difference in estimation of energy and saturated fat intake between Dietplan6 and the diet apps. Estimates of protein and sodium intake were significantly lower using Lose It! (*P*<.001) and FatSecret (*P*=.004 and *P*=.007, respectively) than using Dietplan6. Lose It! also gave significantly lower estimates for other reported outputs (carbohydrate, fat, fiber, and sodium) than did Dietplan6 (*P*<.001, *P*=.003, *P*=.007, and *P*<.001, respectively). S Health (*P*<.001) and MyFitnessPal (*P*=.005, *P*=.002, and *P*=.008, respectively) significantly underestimated calcium, iron, and vitamin C compared with Dietplan6, although there was no significant difference for vitamin A. We observed no other significant differences between Dietplan6 and the apps. Table 1 presents the correlation between estimates of energy and nutrients for Dietplan6 and the apps. Correlation coefficients ranged from *r*=–.12 for iron (S Health vs Dietplan6) to *r*=.91 for protein (FatSecret vs Dietplan6). Noom Coach was limited to energy output, but it had a high correlation with Dietplan6 (*r*=.91). S Health had the greatest variation of correlation, with energy at *r*=.79. Correlations were weakest for iron: S Health (*r*=–.12) and MyFitnessPal (*r*=.13), which were not significant. Correlations between Dietplan6 and both S Health and MyFitnessPal also were not significant for sodium, calcium, iron, vitamin A, and vitamin C.

**Table 1:** Correlation coefficients (*r*) for estimates of energy and nutrient intake between popular diet apps and Dietplan6 using 24-hour weighed food records (n=20)^a^.

Nutrients	Samsung Health	MyFitnessPal	FatSecret	Noom Coach	Lose It!
Energy (kcal)	.79	.85	.86	.91	.87
Carbohydrates (g)	.90^b^	.85	.90	N/A^c^	.66
Protein (g)	.91	.82	.91^b^	N/A	.43^d^
Fat (g)	.84	.91^b^	.83	N/A	.79
Saturated fat (g)	.83^b^	.73b	N/A	N/A	.49^b,d^
Fiber (g)	.70^b^	.70^b^	.66^b^	N/A	.23^b,d^
Sodium (mg)	.44^d^	.44^d^	.47^b,d^	N/A	.51^d^
Calcium (mg)	.37^b,d^	.47^b,d^	N/A	N/A	N/A
Iron (mg)	–.12^d^	.13^b,d^	N/A	N/A	N/A
Vitamin A (μg)	.20^b,d^	.21^b,d^	N/A	N/A	N/A
Vitamin C (mg)	.49^b,d^	.54^b,d^	N/A	N/A	N/A

^a^Correlation assessed using Pearson rank correlation, significant at *P*<.01 (Bonferroni correction applied) (unless otherwise specified).

^b^Correlation assessed using Spearman correlation (*r*_s_), significant at *P*<.01 (unless otherwise specified).

^c^N/A: not applicable.

^d^*P*>.01.

Multimedia Appendix 2 shows the difference, mean values, and limits of agreement between estimates of energy and nutrient intake using the apps compared with the reference (Dietplan6).

**Figure 1:**
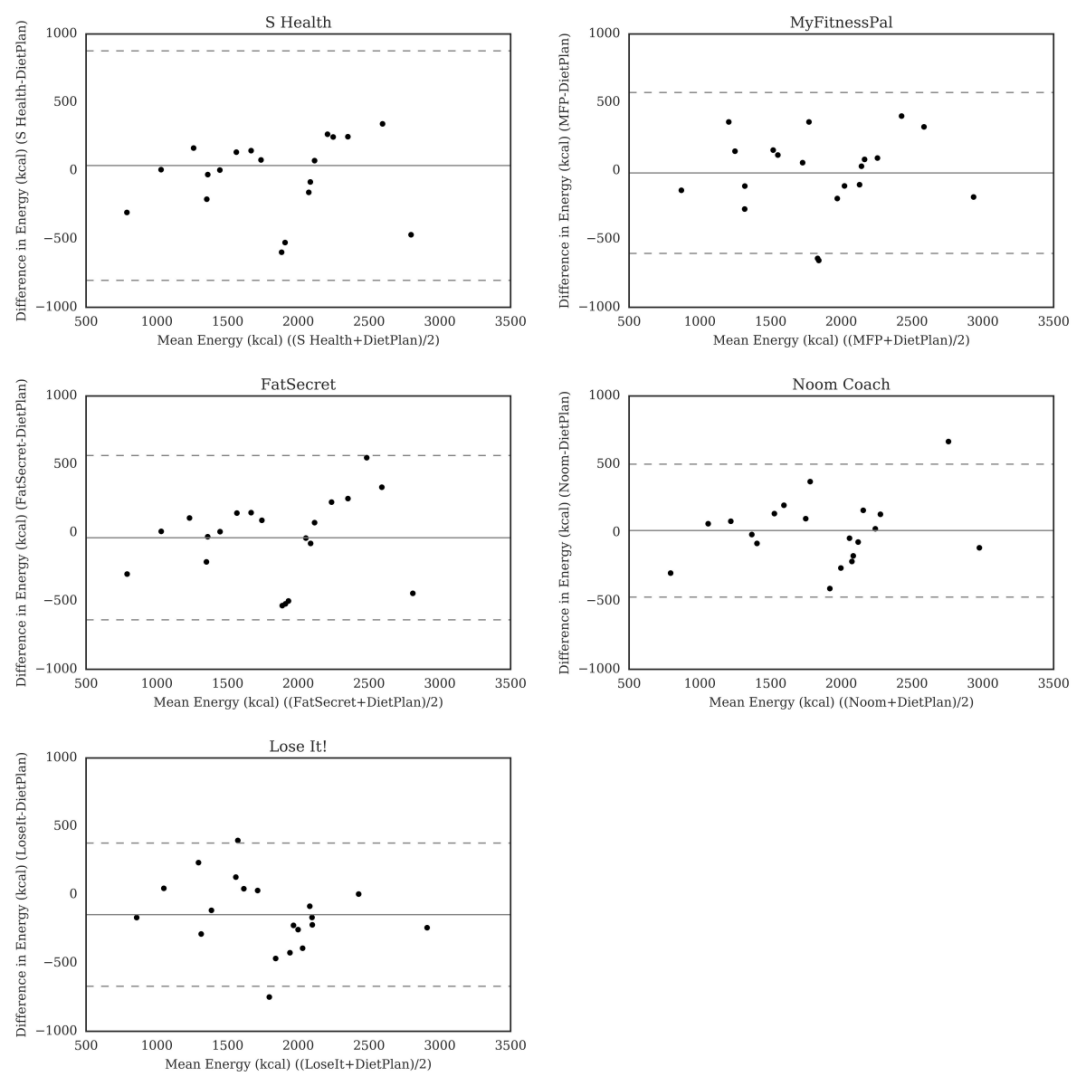
Bland-Altman plots of energy (kcal) difference and average between Samsung Health (S Health), MyFitnessPal (MFP), Fat Secret, Noom Coach, and Lose It! and Dietplan6. The limits of agreement are displayed as 2 SD.

Figure 1 shows Bland-Altman plots for estimates of energy between Dietplan6 and the apps. Overall, less than 5% of cases fell outside of the limits of agreement for estimates of energy using S Health, FatSecret, and Noom Coach compared with Dietplan6, indicating good agreement between the methods. A total of 10% (2/20) of cases fell outside of the limits of agreement for MyFitnessPal and Lose It!, with Lose It! showing the greatest mean difference (bias) in energy compared with Dietplan6 at –146 kcal (Figure 1). The smallest difference in estimated energy was between Noom Coach and Dietplan6 (14.7 kcal). MyFitnessPal, FatSecret, and Lose It! generally reported less energy intake compared with Dietplan6, whereas S Health and FatSecret reported greater intake. The apps with the smallest bias for estimates of nutrients compared with Dietplan6 were as follows: carbohydrate: S Health (5.3 g); protein: S Health (–2.9 g); fat: S Health (–4.6 g); saturated fat: S Health (–3.8 g); fiber: Lose It! (–0.55 g); sodium: S Health (–197 mg); calcium: S Health (–360.9 mg); iron: S Health (–6.1 mg); vitamin A: S Health (180.1 μg); and vitamin C: S Health (–40.3 mg).

**Figure 2:**
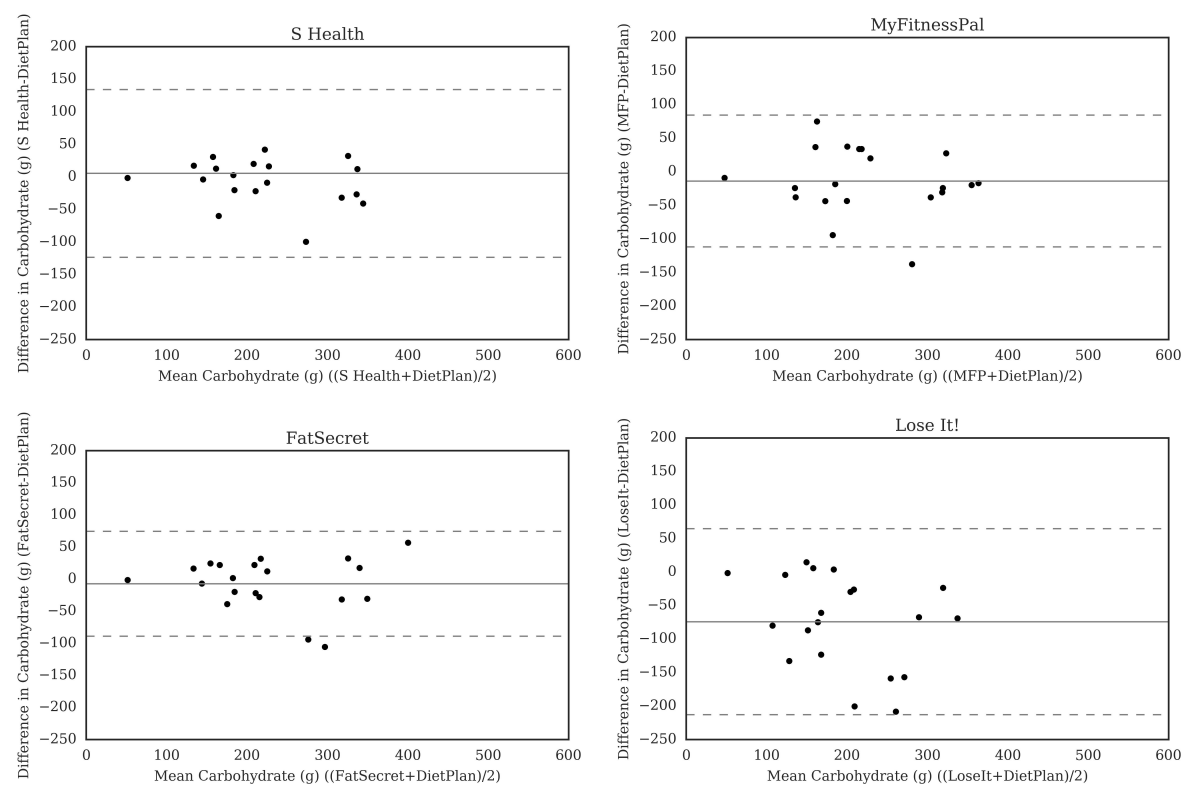
Bland-Altman plots of carbohydrate (g) difference and average between Samsung Health (S Health), MyFitnessPal (MFP), FatSecret, and Lose It! and Dietplan6. The limits of agreement are displayed as 2 SD.

**Figure 3:**
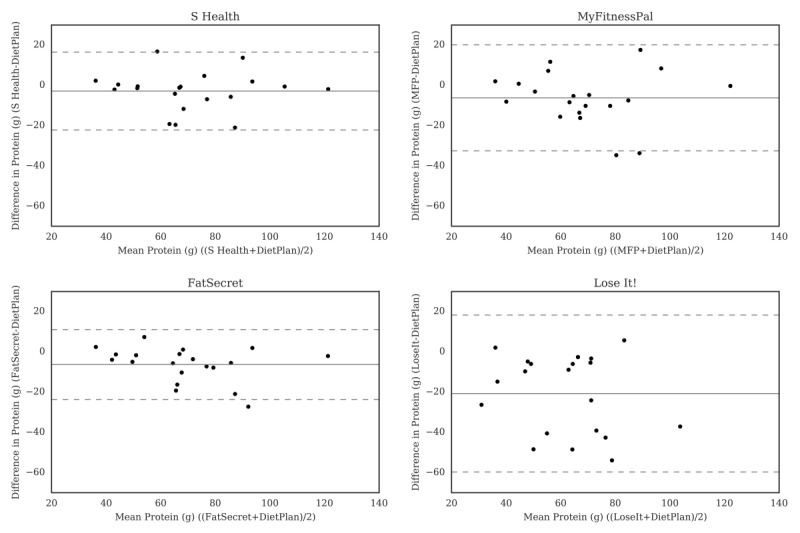
Bland-Altman plots of protein (g) difference and average between Samsung Health (S Health), MyFitnessPal (MFP), FatSecret, and Lose It! and Dietplan6. The limits of agreement are displayed as 2 SD.

**Figure 4:**
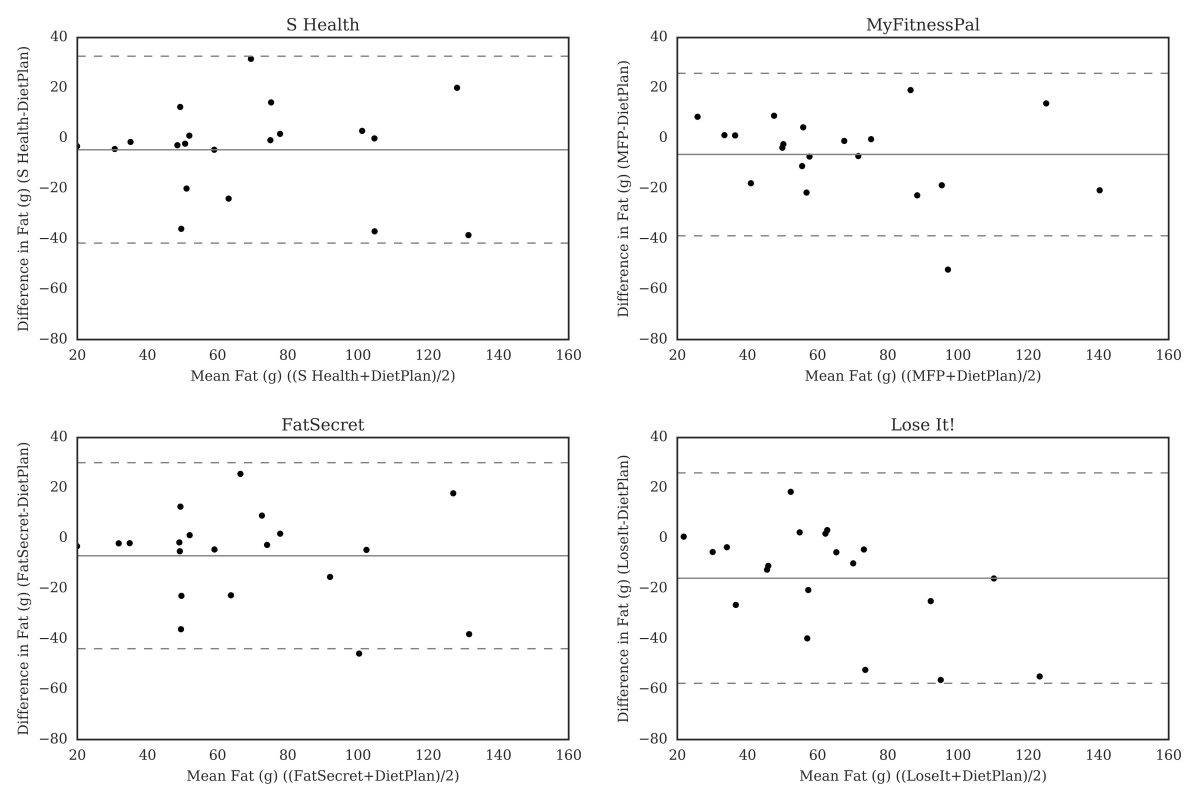
Bland-Altman plots of fat (g) difference and average between Samsung Health (S Health), MyFitnessPal (MFP), FatSecret, and Lose It! and Dietplan6. The limits of agreement are displayed as 2 SD.

**Figure 5:**
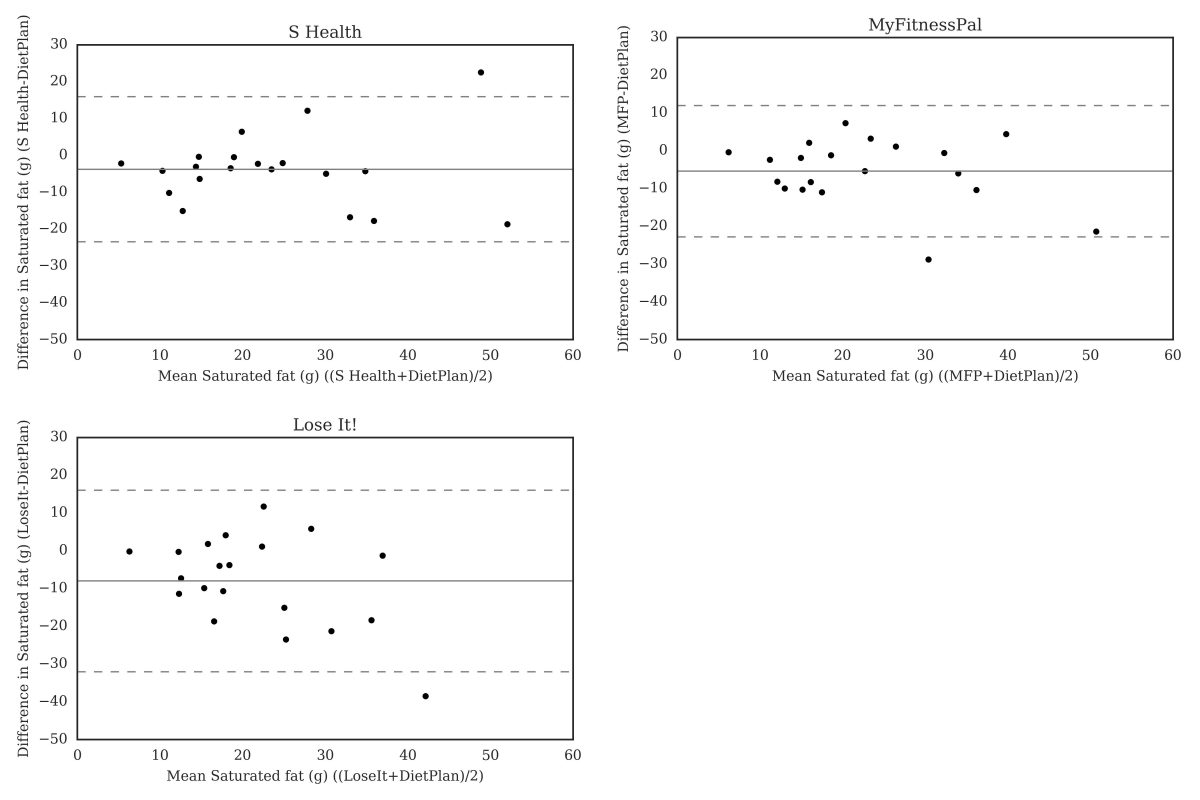
Bland-Altman plots of saturated fat (g) difference and average between Samsung Health (S Health), MyFitnessPal (MFP), and Lose It! and Dietplan6. The limits of agreement are displayed as 2 SD.

**Figure 6:**
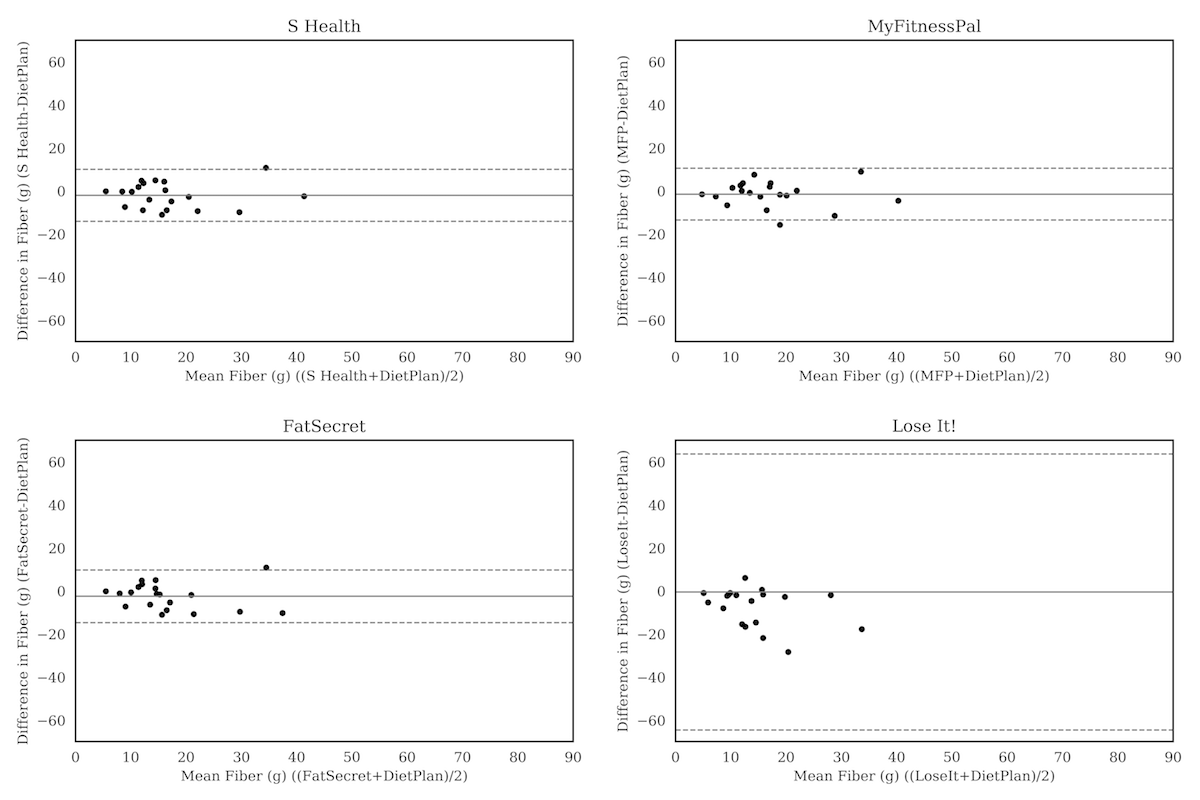
Bland-Altman plots of fiber (g) difference and average between Samsung Health (S Health), MyFitnessPal (MFP), FatSecret, and Lose It! and Dietplan6. The limits of agreement are displayed as 2 SD.

**Figure 7:**
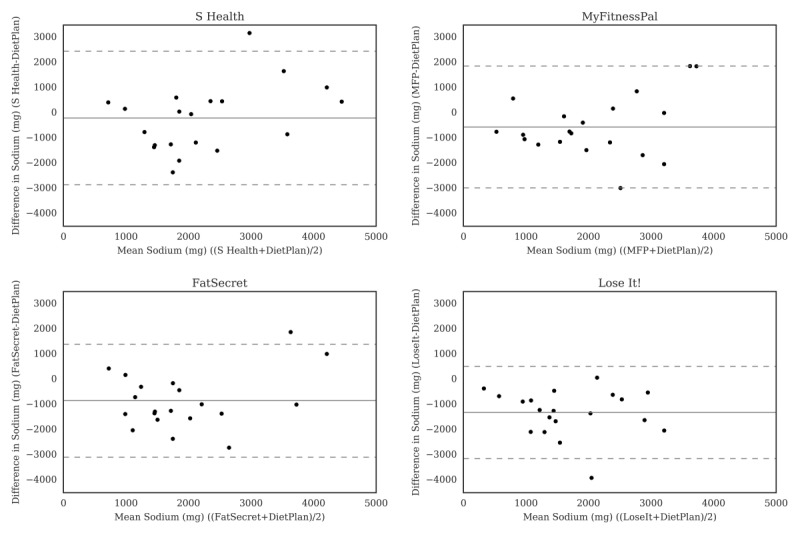
Bland-Altman plots of sodium (mg) difference and average between Samsung Health (S Health), MyFitnessPal (MFP), FatSecret, and Lose It! and Dietplan6. The limits of agreement are displayed as 2 SD.

**Figure 8:**
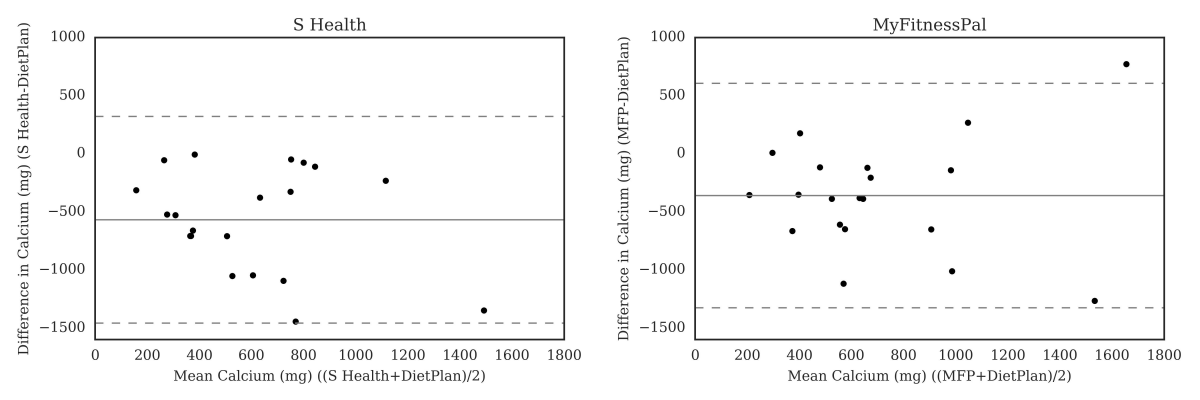
Bland-Altman plots of calcium (mg) difference and average between Samsung Health (S Health) and MyFitnessPal (MFP) and Dietplan6. The limits of agreement are displayed as 2 SD.

**Figure 9:**
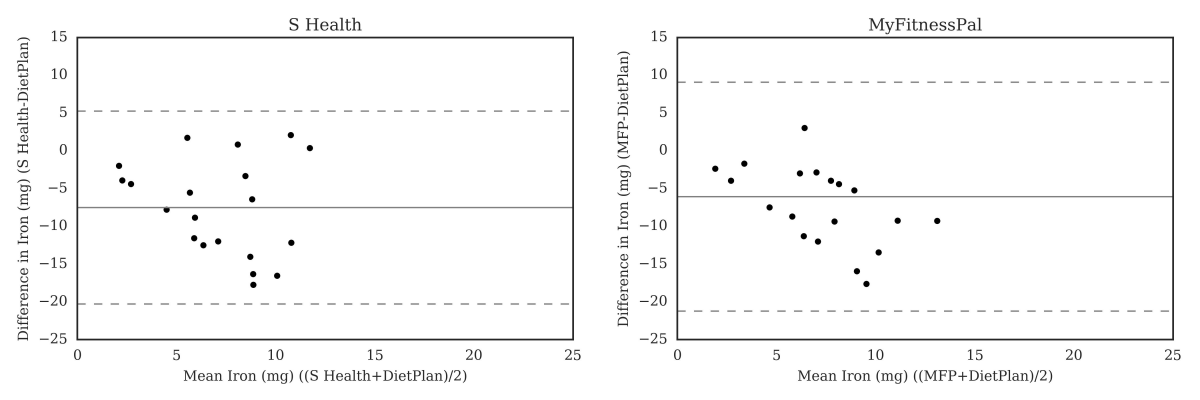
Bland-Altman plots of iron (mg) difference and average between Samsung Health (S Health) and MyFitnessPal (MFP) and Dietplan6. The limits of agreement are displayed as 2 SD.

**Figure 10:**
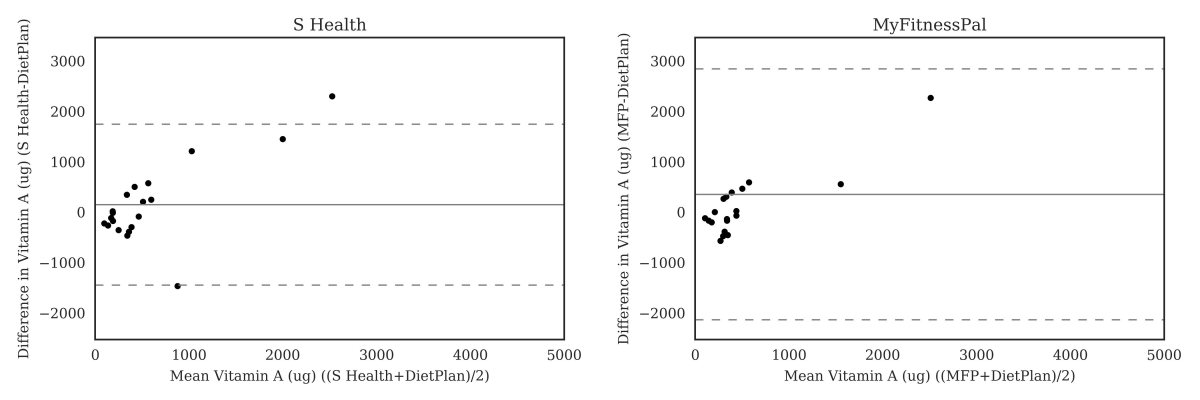
Bland-Altman plots of vitamin A (μg) difference and average between Samsung Health (S Health) and MyFitnessPal (MFP) and Dietplan6. The limits of agreement are displayed as 2 SD.

**Figure 11:**
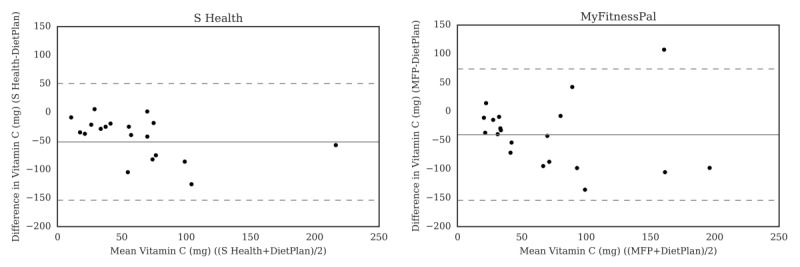
Bland-Altman plots of vitamin C (mg) difference and average between Samsung Health (S Health) and MyFitnessPal (MFP) and Dietplan6. The limits of agreement are displayed as 2 SD.

For carbohydrate (Figure 2), the Bland-Altman plots did not indicate clear proportional bias, although the greatest differences occurred in the range of measurements between 200 and 300 g. The Bland-Altman plots for protein (Figure 3) did not show clear clusters of agreement or disagreement, and the differences seemed to be random fluctuations around the mean. For total fat (Figure 4) and saturated fat (Figure 5), none of the apps had more than 5% of the estimates outside of the limits of agreement and no proportional bias was observed. Lose It! had the broadest limit of agreement for fiber (Figure 6) and all the estimates were within the limits of agreement for this app. We detected no proportional bias for sodium (Figure 7), calcium (Figure 8). or iron (Figure 9). On the other hand, for vitamin A (Figure 10) the data clustered together at low averages, but as the average intakes increased, the difference increased. This suggests a proportional bias in the vitamin A data, as both S Health and MyFitnessPal compared with Dietplan6 showed similar results. For vitamin C, the data clustered together at low averages (Figure 11) and no proportional bias was observed.

## Discussion

### Principal Findings

The primary finding of this study was that popular diet apps are generally comparable in their assessment of energy and key macronutrients (carbohydrate, total fat, and fiber) with a research standard for dietary analysis of WFRs (Dietplan6). However, we observed significant differences for certain apps and nutrients, and Lose It! significantly underestimated 5 of 7 outputs. We found apps to be less able to reliably estimate micronutrients when compared with a widely used and accepted method for dietary analysis.

For estimation of energy intake, S Health, MyFitnessPal, FatSecret, and Noom Coach demonstrated good agreement with Dietplan6, evidenced by small mean differences (14.7 to –36.9 kcal) and strong correlation coefficients (*r*=.79 to *r*=.91). Estimates of carbohydrate and total fat intake were also highly comparable between Dietplan6 and S Health, MyFitnessPal, and FatSecret, with no significant differences between the outputs and similarly small mean differences (5.3 to –13.8 g for carbohydrate and –4.6 to –15.9 g for total fat). Raatz et al also found no significant differences in estimates of energy, carbohydrate, and total fat when comparing nutrient analysis of a 3-day WFR using the Tap & Track app and US Department of Agriculture (USDA) nutrient analysis program (mean differences 85 kcal, 15.4 g, and 2.6 g, respectively) [9]. However, in contrast to our study, in the study of Raatz and colleagues, users (n=19) entered the WFR into Tap & Track and a registered dietitian entered data into the USDA nutrient analysis program [9]. The authors suggested that the observed variability in Bland-Altman plots between methods may have been associated with the ability of the users to pick appropriate food items and ensure that all data were recorded [9,15], which would not have been an issue in our study, as a single researcher entered data into all apps and the reference program.

Protein intake was significantly underestimated by FatSecret and Lose It! compared with Dietplan6, and correlations between the methods varied from *r*=.43 to *r*=.91. This indicates that these apps are less reliable at estimating protein intake than at estimating energy, carbohydrate, and total fat intake. Tap & Track’s estimate of protein intake did not differ from the USDA nutrient analysis program output [9] and, in general, apps comparing traditional paper-based (eg, 24-hour recall, WFR) versus electronic-based methods of estimating energy intake and macronutrients have found comparable and acceptable results [6,16,17].

Correlations were weaker between S Health, MyFitnessPal, and FatSecret and Dietplan6 for sodium (*r*=.44 to *r*=.51), with significantly lower estimates given by FatSecret. S Health and MyFitnessPal were the only apps to assess iron, calcium, vitamin A, and vitamin C, which we observed were significantly underestimated by the apps (*r*=–.12 to *r*=.54). While there was no significant difference in estimated vitamin A between these apps and Dietplan6, results of the Bland-Altman analysis revealed a proportional bias, whereby higher intakes of vitamin A were less well estimated by the apps. These data suggest that the apps did not reliably estimate micronutrient intake in comparison with the reference method.

In this study, a single researcher was responsible for entering the WFRs into each of the methods (apps and Dietplan6) and, therefore, discrepancies in outputs were most likely due to variations in the apps’ nutrient databases, as opposed to variations in data entry. For example, apps may use different sources to compile nutrient databases, including open source and manufacturer databases, and some apps involve users in the expansion of their database, which may result in inaccuracies and incomplete data [12]. The observation that micronutrients were less reliably estimated by the apps, compared with macronutrients, may be attributed to the fact that micronutrients are declared only voluntarily on nutritional labels of foods, and therefore apps using commercial databases are more likely to have missing composition data [18]. In addition, 4 of the 5 apps studied (ie, excluding S Health) have a barcode feature [10], which relies on nutrition data provided by manufacturers, which may have incomplete micronutrient data. This method is less reliable than the chemical analysis done in most of the food databases used by the reference method (McCance and Widdowson’s nutrient database [13]).

For those apps unable to reliably assess intake of particular nutrients (eg, protein, sodium, vitamin A), the display of the data to the user is potentially misleading. For example, underestimation of sodium intake for a patient with high blood pressure (who would typically be advised to reduce their salt intake) may result in the user unknowingly exceeding their recommended intake. Contrary to medical devices, apps available in app stores are not regulated by the US Food and Drug Administration. The level of validity of the nutrition-related apps available for commercial download is often uncertain, creating ambivalence for health organizations about their use. One reason for this is the absence of a standard method of assessing the validity of health-related apps [8]. Given the lack of transparency in the source of nutritional data used by app developers, it will be important to repeat these assessments in the future. With the increase in awareness of the possibilities of app-based dietary assessment and use of the most popular nutrition-related apps, our findings will help clinicians and researchers to be better informed about using these apps to facilitate nutrition care and research, and about whether their use can replace traditional research software. Reported barriers to app use in dietetic practice in the 3-country survey by Chen et al included lack of awareness about the best app to recommend [7] and, while MyFitnessPal was the most popular app recommended by dietitians in this survey, none of the other apps assessed in our study were recommended [7].

### Strengths and Limitations

Strengths of this study include the use of a WFR to assess dietary intake and the entry of WFR data (reference method and apps) by a single researcher. Whereas previous studies have compared user versus professional entry of the same record [9], or user versus professional entry of records taken on separate occasions [19], this study eliminated the biases associated with user entry (eg, selection of an appropriate food item, estimation of portion size [15]). The strengths of using a WFR, as opposed to a 24-hour recall, are that dietary intake is recorded prospectively (ie, at the time of intake) and users are prompted to provide the level of detail required for analysis (eg, describe the brand, name, weight, and leftovers of each item). Prospective WFR recording potentially provides a greater level of detail than does a 24-hour recall, which is a retrospective method that relies on the participant’s memory, as well as the skill of the researcher-interviewer to obtain sufficient detail on each food item [20].

Limitations include a relatively small sample size (n=20) and the use of 24-hour WFRs collected prior to the study. For example, it was not possible to check ambiguous entries (eg, unspecified brand) with the participant, although where we made assumptions, these were verified by an independent researcher and applied across all methods. Exploration of the impact of, for example, training on the accuracy of dietary data input by users would provide further evidence for the use of popular nutrition apps in the research and clinical setting.

### Conclusions

This study compared the accuracy of the outputs of 5 nutrition-related apps (S Health, MyFitnessPal, FatSecret, Noom Coach, and Lose It!) against a research standard, using 24-hour WFRs as input. The findings suggest that the apps provide estimates of energy and saturated fat intake that are comparable with Dietplan6. With the exception of Lose It!, the apps also provided comparable estimates of carbohydrate, total fat, and fiber. Two apps displayed a tendency to underestimate protein and sodium (FatSecret and Lose It!). The estimates of micronutrient intake (calcium, iron, vitamin A, and vitamin C) by 2 apps (S Health and MyFitnessPal) were inconsistent and less reliable. Overall, the nutritional outputs provided by S Health and Noom Coach, which provided only energy, were the most reliable for this output. As the use and availability of nutrition-related apps grow, there are increasing opportunities for apps to support research in nutrition, care, and self-management of noncommunicable diseases. This study highlighted in which aspects the outputs from these apps are comparable with, and where they differ significantly from, widely accepted and professionally used nutrition analysis software, thereby making a valuable contribution toward helping clinicians and researchers to make better-informed decisions about using these apps in research and practice.

## Appendix

Multimedia Appendix 1 Mean estimates of energy and nutrient intake.

Multimedia Appendix 2 Difference and Bland-Altman limits of agreement.

## Abbreviations

S Health: Samsung Health
USDA: US Department of Agriculture
WFR: weighed food record
