# Supplementary material for: Popular Nutrition-Related Mobile Apps: An Agreement Assessment Against a UK Reference Method
Source: JMIR Mhealth Uhealth. 2019 Feb 20;7(2):e9838. doi: 10.2196/mhealth.9838 (PMC6401676; doi:10.2196/mhealth.9838)
Supplement: Multimedia Appendix 1 [file mhealth_v7i2e9838_app1.docx]

Mean estimates of energy and nutrient intake between popular diet apps and DietPlan6 using 24-hour weighed food records (n=20)^a^.

|  | DietPlan6 | | Diet apps | | | | | | | | | | | | | | |
| --- | --- | --- | --- | --- | --- | --- | --- | --- | --- | --- | --- | --- | --- | --- | --- | --- | --- |
|  |  |  | S Health | | | MyFitnessPal | | | FatSecret | | | Noom Coach | | | Lose It! | | |
| Nutrients | Mean (SD) | Median (IQR^b^) | Mean (SD) | Median (IQR) | *P* value | Mean (SD) | Median (IQR) | P value | Mean (SD) | Median (IQR) | *P* value | Mean (SD) | Median (IQR) | *P* value | Mean (SD) | Median (IQR) | *P* value |
| Energy (kcal) | 1850 (527) | 2076 (718) | 1886 (682) | 1757 (925) | .70 | 1832 (545) | 1812 (691) | .79 | 1813 (580) | 1710 (776) | .59 | 1864 (578) | 1917 (584) | .79 | 1704 (456) | 1728 (482) | .022 |
| Carbohydrate (g) | 232.3 (91.3) | 210.1 (150.2) | 237.6 (117.3) | 219.3 (135.4) | .91^c^ | 218.6 (84.6) | 215.5 (119.8) | .22 | 225.1 (88.6) | 210.7 (85.9) | .44 |  |  |  | 158.3 (71.4) | 156.7 (81.5) | *<.001* |
| Protein (g) | 72.3 (22.4) | 73.2 (32.5) | 69.5 (22.0) | 66.6 (26.4) | .20 | 66.1 (21.3) | 62.0 (15.0) | .049 | 66.0 (19.7) | 64.4 (21.2) | *.004*^c^ |  |  |  | 51.6 (18.4) | 52.6 (23.0) | *<.001* |
| Fat (g) | 71.1 (33.2) | 64.4 (31.6) | 66.5 (31.8) | 56.1 (39.9) | .29 | 64.6 (29.0) | 56.0 (28.0) | .12^c^ | 64.1 (29.8) | 56.2 (34.3) | .11 |  |  |  | 55.2 (22.4) | 58.8 (26.8) | *.003* |
| Saturated fat (g) | 25.6 (13.2) | 21.1 (17.1) | 21.8 (13.6) | 21.1 (14.6) | .03^c^ | 20.1 (10.8) | 17.5 (16.5) | .012^c^ |  |  |  |  |  |  | 17.6 (8.5) | 16.6 (11.2) | .014^c^ |
| Fiber (g) | 18.0 (9.4) | 15.8 (10.9) | 15.9 (9.5) | 14.2 (7.5) | .16^c^ | 16.4 (9.0) | 14.0 (7.3) | .35^c^ | 15.4 (8.5) | 14.2 (6.0) | .08^c^ |  |  |  | 17.4 (30.6) | 9.0 (10.8) | *.007*^c^ |
| Sodium (mg) | 2355 (966) | 2215 (1043) | 2158 (1432) | 1796 (1942) | .52 | 1800 (1271) | 1435 (1257) | .055 | 1513 (1263) | 1096 (894) | *.007*^c^ |  |  |  | 1044 (870) | 655 (1806) | *<.001* |
| Calcium (mg) | 885.4 (436.3) | 830.5 (399.3) | 315.8 (340.6) | 163.3 (585.0) | *<.001*^c^ | 524.5 (466.2) | 445.0 (335.0) | *.005*^c^ |  |  |  |  |  |  |  |  |  |
| Iron (mg) | 10.9 (4.7) | 11.0 (5.5) | 3.4 (3.9) | 1.6 (5.2) | *<.001* | 4.8 (6.3) | 3.3 (5.0) | *.002*^c^ |  |  |  |  |  |  |  |  |  |
| Vitamin A (μg) | 491.8 (420.8) | 400.4 (317.2) | 671.9 (965.7) | 232.0 (572.9) | .55^c^ | 875.5 (1583.6) | 352.5 (513.8) | .48^c^ |  |  |  |  |  |  |  |  |  |
| Vitamin C (mg) | 89.8 (62.2) | 73.0 (68.3) | 38.0 (41.0) | 32.2 (31.2) | *<.001*^c^ | 49.5 (55.2) | 27.6 (38.7) | *.008*^c^ |  |  |  |  |  |  |  |  |  |

^a^Normally distributed data analyzed using paired *t* tests (unless otherwise specified), significant at P<.01 (Bonferroni correction applied).

^b^IQR: interquartile range.

^c^Nonnormal data analyzed using Wilcoxon signed-rank tests, significant at *P*<.01.
