# Supplementary material for: Popular Nutrition-Related Mobile Apps: An Agreement Assessment Against a UK Reference Method
Source: JMIR Mhealth Uhealth. 2019 Feb 20;7(2):e9838. doi: 10.2196/mhealth.9838 (PMC6401676; doi:10.2196/mhealth.9838)
Supplement: Multimedia Appendix 2 [file mhealth_v7i2e9838_app2.docx]

Difference and Bland-Altman limits of agreement (LOA) for estimated energy and nutrient intake between popular diet apps and DietPlan6 using 24-hour weighed food records (n=20).

| Nutrients | S Health vs DietPlan6 | | | | MyFitnessPal vs DietPlan6 | | | | FatSecret vs DietPlan6 | | | | Noom Coach vs DietPlan6 | | | | Lose It! vs DietPlan6 | | | |
| --- | --- | --- | --- | --- | --- | --- | --- | --- | --- | --- | --- | --- | --- | --- | --- | --- | --- | --- | --- | --- |
|  | Bias | SD | LOA | % LOA^a^ | Bias | SD | LOA | % LOA | Bias | SD | LOA | % LOA | Bias | SD | LOA | % LOA ^a^ | Bias | SD | LOA | % LOA |
| Energy (kcal) | 36.7 | 420 | –803, 876 | 100 | –17.4 | 294 | –606, 571 | 90 | –36.9 | 300 | –637, 563 | 100 | 14.7 | 243 | –472, 501 | 100 | –146.0 | 262 | –670, 378 | 90 |
| Carbohydrate (g) | 5.3 | 64.5 | –124, 134 | 100 | –13.8 | 49.1 | –112, 84 | 95 | –7.2 | 40.8 | –89, 75 | 90 |  |  |  |  | –74.1 | 69.4 | –213, 65 | 100 |
| Protein (g) | –2.9 | 9.7 | –22, 16 | 96 | –6.2 | 13.2 | –33, 20 | 90 | –6.3 | 8.7 | –24, 11 | 95 |  |  |  |  | –20.7 | 19.5 | –60, 18 | 100 |
| Fat (g) | –4.6 | 18.5 | –42, 33 | 100 | –6.5 | 16.1 | –39, 26 | 95 | –7.0 | 18.5 | –44, 30 | 95 |  |  |  |  | –15.9 | 20.9 | –58, 26 | 100 |
| Saturated fat (g) | –3.8 | 9.9 | –24, 16 | 95 | –5.4 | 8.7 | –23, 12 | 95 |  |  |  |  |  |  |  |  | –8.0 | 12.0 | –32, 16 | 95 |
| Fiber (g) | –2.0 | 6.1 | –14, 10 | 95 | –1.5 | 6.0 | –14, 11 | 95 | –2.5 | 6.1 | –15, 10 | 95 |  |  |  |  | –0.55 | 32.1 | –65, 64 | 100 |
| Sodium (mg) | –197 | 1326 | –2850, 2456 | 95 | –555 | 1211 | –2977, 1867 | 95 | –842 | 1123 | –3088, 1405 | 95 |  |  |  |  | –1310 | 917 | –3143, 523 | 95 |
| Calcium (mg) | –360.9 | 482.7 | –1418, 1778 | 100 | –569.6 | 444.5 | –1458, 319 | 95 |  |  |  |  |  |  |  |  |  |  |  |  |
| Iron (mg) | –6.1 | 7.6 | –21, 9 | 100 | –7.5 | 6.4 | –20, 5 | 100 |  |  |  |  |  |  |  |  |  |  |  |  |
| Vitamin A (μg) | 180.1 | 799.0 | –1418, 1778 | 90 | 383.7 | 1245.3 | –2016, 2874 | 100 |  |  |  |  |  |  |  |  |  |  |  |  |
| Vitamin C (mg) | –40.3 | 57.0 | –154, 74 | 100 | –51.8 | 50.9 | –154, 50 | 95 |  |  |  |  |  |  |  |  |  |  |  |  |

^a^Percentage of cases within the LOA.
